# Implementation of a children’s hospital-wide central venous catheter insertion and maintenance bundle

**DOI:** 10.1186/1472-6963-13-417

**Published:** 2013-10-14

**Authors:** Onno Helder, René Kornelisse, Cynthia van der Starre, Dick Tibboel, Caspar Looman, René Wijnen, Marten Poley, Erwin Ista

**Affiliations:** 1 Department of Pediatrics, Division of Neonatology, Erasmus MC - Sophia Children’s Hospital, Erasmus University Medical Centre, Rotterdam, The Netherlands; 2 Department of Pediatric Surgery, Erasmus MC - Sophia Children’s Hospital, Erasmus University Medical Centre, Rotterdam, The Netherlands; 3 Department of Public Health, Erasmus University Medical Centre, Rotterdam, The Netherlands; 4 Institute for Medical Technology Assessment (iMTA), Erasmus University Rotterdam, Rotterdam, The Netherlands

**Keywords:** Bloodstream infection, Central venous catheter, Prevention, Children’s hospital, Implementation, Education

## Abstract

**Background:**

Central venous catheter-associated bloodstream infections in children are an increasingly recognized serious safety problem worldwide, but are often preventable. Central venous catheter bundles have proved effective to prevent such infections. Successful implementation requires changes in the hospital system as well as in healthcare professionals’ behaviour. The aim of the study is to evaluate process and outcome of implementation of a state-of-the-art central venous catheter insertion and maintenance bundle in a large university children’s hospital.

**Methods/design:**

An interrupted time series design will be used; the study will encompass all children who need a central venous catheter. New state-of-the-art central venous catheter bundles will be developed. The Pronovost-model will guide the implementation process. We developed a tailored multifaceted implementation strategy consisting of reminders, feedback, management support, local opinion leaders, and education. Primary outcome measure is the number of catheter-associated infections per 1000 line-days. The process outcome is degree of adherence to use of these central venous catheter bundles is the secondary outcome. A cost-effectiveness analysis is part of the study. Outcomes will be monitored during three periods: baseline, pre-intervention, and post-intervention for over 48 months.

**Discussion:**

This model-based implementation strategy will reveal the challenges of implementing a hospital-wide safety program. This work will add to the body of knowledge in the field of implementation. We postulate that healthcare workers’ willingness to shift from providing habitual care to state-of-the-art care may reflect the need for consistent care improvement. Trial registration: Dutch trials registry, trial # 3635.

**Trial registration:**

Dutch trials registry (http://www.trialregister.nl), trial # 3635

## Background

Catheter-associated bloodstream infections (CA-BSIs) in hospitals are a worldwide serious persistent problem. Although often preventable, they are a source of morbidity, mortality, prolonged hospital stay, and rising costs [1-4]. CA-BSIs notably occur in units where many patients have central venous catheters (CVC); reported figures range from 1.2 to 23.0 CA-BSIs per 1000 line-days in neonatal intensive care units and from 1.8 to 7.8 CA-BSIs per 1000 line-days in pediatric intensive care units [3-8]. The corresponding figures from a pilot study we performed in 2011 are 11.2 and 7.9. Both unit types admit patients with compromised immune system and patients undergoing invasive procedures [9]. High incidences in neonatal intensive care units may be due to an immature host defence in preterm infants [5,10].

Pronovost et al. showed that the introduction of improved CVC insertion techniques helped to bring down the incidence of CA-BSIs to nearly zero 16 to 18 months later. This study was mainly in adult intensive care units, but the successful outcome encouraged us to improve the CVC care for all children admitted to our children’s hospital [11]. A model developed by Pronovost and colleagues (“Pronovost-model”) will guide the current study protocol [12].

There is growing evidence that a combination of interventions, termed CVC insertion and maintenance bundles, may be effective in preventing CA-BSIs in infants and children as well [9,13-15]. For example by ensuring maximum sterile barrier during CVC insertion and appropriate disinfection during intravenous medication administration. Regrettably, the evidence is limited by differences in the bundle’s insertion components in the various studies [13,16].

Each ward in our children’s hospital tends to have its own habits and protocols with regard to CVC insertion and maintenance care. These protocols are not all in line with the available evidence for optimal CVC care. In addition, the inconsistent policies may confuse patients and their parents upon transfer to another ward. It would be best, therefore, to have one hospital-wide, state-of-the-art CVC insertion and maintenance protocol. Little has been published, however, on successful implementation of large scale innovations like this in a children’s hospital, let alone one guided by the Pronovost-model. The proposed study aims to obtain solid evidence for an effective implementation method. Furthermore, cost-effectiveness of the implementation of a CVC bundle has hardly been addressed, so the question remains whether it provides good value for money. CA-BSIs are known to be associated with high costs. A study in a neonatal intensive care unit in Belgium found that children with CA-BSIs stayed a mean 24 days longer in hospital at a mean extra charge of approximately €12,000 (2). In another study from a pediatric intensive care unit in the USA, the corresponding figures were 9 days and $33,000 (4). Concrete evidence about the potential of a CVC bundle to lower the costs is still lacking however. This study may help enlarge the body of evidence on cost-effectiveness.

The objectives are fourfold: (1) to assess the effects of hospital-wide implementation of a CVC insertion and maintenance bundle on the incidence of CA-BSIs per 1000 line-days over 48 months; (2) to assess adherence to use of the CVC insertion bundle; (3) to assess adherence to use of the maintenance bundle; and (4) to explore the cost-effectiveness of implementing a CVC insertion and maintenance protocol.

### Scientific hypothesis

We will test the following hypothesis: implementation of hospital-wide CVC insertion and maintenance bundles on the guidance of the Pronovost-model promotes protocol adherence and reduces the number of CA-BSIs.

## Methods/design

### Study design

**Figure 1:**
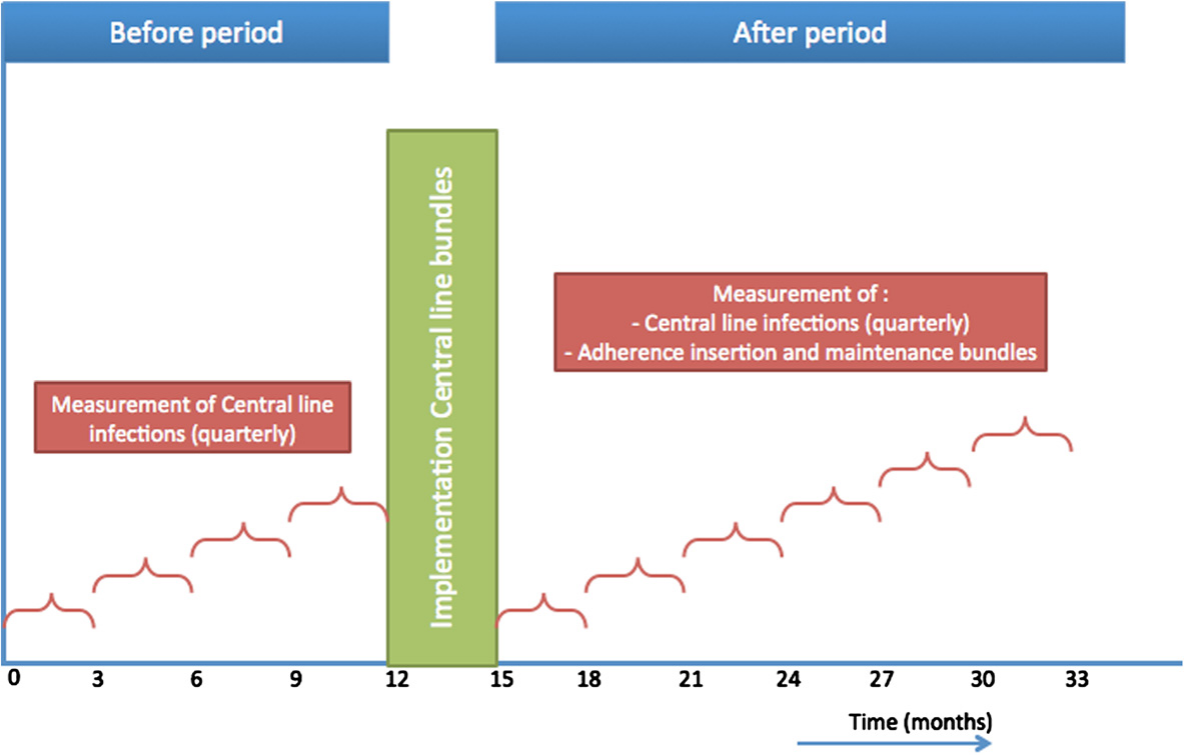
Interrupted time series study design showing the 48-month timeline of the study, divided into pre-intervention, intervention, and post-intervention stage.

An interrupted times series (ITS) analysis will be performed covering a number of three-month periods in three subsequential phases: pre-intervention, intervention, and post-intervention (Figure 1). The Outbreak Reports and Intervention studies of Nosocomial infection (ORION) statement [17] will be applied to guarantee quality of reporting and the use of appropriate statistical techniques.

### Setting and participants

The setting of this study is the Erasmus MC-Sophia Children’s Hospital, Rotterdam, the Netherlands. In 2011 it counted 252 beds, 12,403 admissions, and 53,541 patient-days. Distribution over the various wards was as follows: Neonatal intensive care unit: 27 beds, 775 admissions (8,447 patient-days); pediatric intensive care unit: 34 beds (including 6 beds in high dependency transfer unit), 1504 admissions (10,070 patient-days); medium care units: 96 beds, 4,536 admissions (29,436 patient-days); day-care ward, 14 beds, 5,588 admissions (5,588 patient-days); and operation room suite: 8 operation rooms, and 9,207 surgeries. All wards except the day-care ward will be participating in this study.

### Implementation model

Implementation will be guided by the Pronovost-model [12]. The model gives priority to systems operation, centralised support, a collaborative culture, but also promotes local responsibility. Four stages are distinguished: (1) Collect evidence on interventions associated with improved outcomes and select the best feasible ones; (2) Identify possible barriers to implementation by monitoring the current practice and asking stakeholders why they would not comply with current protocols. On the other hand, identify intrinsic and extrinsic motivators that could help implementation; (3) Select process or outcome measures for adherence to the new protocols. Monitoring will encourage healthcare workers’ desired behaviour; (4) All local healthcare workers as much as possible provide care according to the new protocols. Striving to achieve an overall high adherence, implementation will be executed by means of the 'four Es’: 'engage’, 'educate’, 'execute’, and 'evaluate’.

#### ***Collecting evidence***

A state-of-the-art CVC protocol should meet the Centers for Disease Control and Prevention (CDC) recommendations, be in line with leading studies [3,13], and be acceptable for key players in all wards. It must ensure that children who are moved internally always receive the same CVC care. We will set up a working group and a steering committee for this purpose. The working group will first make an inventory of all CVC protocols including port catheters currently used in our children’s hospital. Evidence for effective measures to prevent infections will be retrieved from relevant published studies (Table 1) and assessed on criteria established by the Dutch Institute for Healthcare Improvement CBO in collaboration with the Dutch Cochrane library [18]. The most frequently mentioned measures in these studies are maximum sterile barrier during insertion procedure, site cleaning with chlorhexidine, maximum aseptic administration of intravenous medication, promotion of hand hygiene, and daily evaluation of CVC indication [14,19-22]. Based on the flaws in current practice observed in a pilot study we selected 10 interventions that seemed most appropriate for our patients. The key features are listed in Table 2. In case the literature is inconclusive consensus will be reached through discussion.

**Table 1:** Overview of interventions in and effectiveness of CVC bundles in neonatal intensive care unit and pediatric intensive care unit settings

**Author (year)**	**Setting**	**Design**	**Intervention**	**Reduction of CA**-**BSI**	**Level of evidence**
Wirtschafter et al. (2010)	NICU	A	Proper CVC insertion, hand hygiene promotion, closed tubing system, improved hub care	From 4.32 to 3.22 per 1000 line-days	2-
Sannoh et al. (2010)	NICU	B	Hand hygiene promotion, proper hub care using chloorhexedine with alcohol, glove use promotion, CVC documentation	From 23 to 12 per 1000 line-days*	2++
Bizzarro et al. (2010)	NICU	B	Proper CVC placement, promotion of hand hygiene, daily evaluation CVC need, infection surveillance, dressing replaced on indication	From 8.40 to 1.28 cases per 1000 line-days*	2+
Andersen et al. (2005)	NICU^#^	B	Hand hygiene promotion, maximum barrier during CVC insertion, daily evaluation need for CVC removal	From 21% to 9% (P=0.05, confidence intervals 0.19–1.0)*	2+
Costello et al. (2008)	PICU	C	Hand hygiene promotion, daily evaluation need for CVC removal, CVC insertion kid	From 7.8 to 4.7 and to 2.3 per 1000 line-days	2-
McKee et al. (2008)	PICU	D	Proper insertion and nursing care, empower nurses to stop the insertion procedure if guidelines were not followed, using a checklist to ensure adherence to the guidelines, providing weekly performance feedback, promotion of hand hygiene, chlorhexedine skin preparation,	From 5.2 to 3.0 per 1000 line-days*	2+
Jeffries et al. (2009)	PICU	E	Maximum sterile barrier during CVC insertion, hand hygiene promotion, apply transparent dressing, prepare skin with anti and/ or detergent chlorhexidine gluconate 2%,	From 6.3 to 4.3 per 1000 line-days*	2+
Miller et al. (2010)	PICU	F	Disinfect catheter site using chlorhexedine, maximum barrier, full barrier during pre-packages of the insertion tray, daily assess CVC need, gauze change every 2 days	From 5.4 to 3.1 per 1000 line-days*	2+
Wheeler et al. (2011)	Children’s hospital-wide	D	Full barrier precautions, chlorhexedine skin preparation with 2 minutes scrub and 1 minute air dry, use of insertion checklist, staff empowerment to stop the insertion procedure, daily assess CVC need, promotion of hand hygiene, chlorhexidine-impregnated sponge placed at insertion site, glove use for all CVC manipulations, change dressing every 7 day or on indication earlier, replace tubing sets no more than 72 hour, cap change every 7 day	From 3 to <1 per 1000 line-days*	2+
Chuengchitraks et al. (2010)	PICU	G	Promotion of hand hygiene, maximal barrier precautions, provide skin antiseptic, optimal catheter site selection	From 2.6 to 2.4 per 1000 line-days	2-

Design: A: multi center prospective pre-test posttest study; B: single center prospective pretest and posttest study; C: single center retrospective pretest and prospective posttest group, interrupted time series design; D: single center retrospective pretest and prospective posttest group, time series design; E: multicenter, prospective pretest and posttest group, time series; F: multicenter retrospective pretest and posttest group, interrupted time series; G: single center cohort study; 1++ High-quality meta-analyses, systematic reviews of RCTs, or RCTs with a very low risk of bias; 1+ Well-conducted meta-analyses, systematic reviews of RCTs, or RCTs with a low risk of bias; 1- Meta-analyses, systematic reviews of RCTs, or RCTs with a high risk of bias; 2++ High-quality systematic reviews of case–control or cohort studies, or high-quality case–control or cohort studies with a very low risk of confounding, bias, or chance and a high probability that the relationship is causal; 2+ Well-conducted case–control or cohort studies with a low risk of confounding, bias, or chance and a moderate probability that the relationship is causal; 2- Case–control or cohort studies with a high risk of confounding, bias or chance, and a significant probability that the relationship is not causal; 3 Non-analytic studies; for example, case reports, case series; 4 Expert opinion.

*CVC* central venous catheter, *BSI* bloodstream infection, *NICU* neonatal intensive care unit, *PICU* pediatric critical care unit.

^#^Premature born infants with birth weight < 1500 grams; and *indicate significant reduction of bloodstream infections per 1000 line-days.

**Table 2:** Major components of CVC insertion bundle and maintenance bundle in The Erasmus MC-Sophia Children’s Hospital

**Insertion bundle**	**Components**
Strict hand hygiene according to “my 5 moments”^#^	
Full sterile barrier	Hygienic precaution training for all healthcare workers who insert CVCs
	Sterile gown, mask, hat, double sterile gloves worn by the healthcare worker who inserts the CVC
	Use of pre-packaged CVC insertion kit
	Sterile drape covering the patient for least 80%
Physical barrier between the environment and the child, insertion cart, and healthcare worker who inserts the CVC	
Insertion site cleaned with alcohol 70% with chlorhexidine 0.5% (chlorhexedine 0.2% in water for infants < 26 weeks gestational age)	
Use of a timer displayed at the bedside’s screen to secure an air dry time of at least 30 seconds	
Use a new needle after each attempt to insert a CVC through the intact skin	
Chlorhexedine impregnated sponge attached at insertion site (not in infants < 40 weeks gestational age)	
**Maintenance bundle**	
Strict hand hygiene according to “my 5 moments”^#^	
Hand hygiene	Hand washing when entering the unit, after using the bathroom, and when hands are visually soiled
	Hand disinfection during all other occasions
	Using gloves when risk of contact with body fluids
Catheter site care	Daily inspection of insertion site (e.g. redness, collection of fluids, swelling)
	Change transparent semi permeable dressing on indication only (e.g. loosened, collection of fluids)
Hub care	Disinfection of the hub with alcohol 70% and air dry for at least 30 seconds
	Use of a timer displayed at the bedside’s screen to secure an air dry time of at least 30 seconds
Tubing care	Replace continuous tubing sets every 96 hours, unless visually soiled of contaminated
	Daily replace tube containing lipids, medication, blood or blood products
	Remove intermitted administration sets directly after administration of medication
Daily question whether CVC still needed	

^#^My 5 moments adopted from the CDC [23].

*CVC* central venous catheter.

The working group will prepare a draft protocol for approval by the steering group. This will encompass both the insertion bundle and the maintenance bundle, each with its sub themes. The insertion bundle consists of: preparing a CVC insertion cart and providing assistance to the physician or nurse practitioner during the CVC insertion procedure (e.g. PICC line, umbilical catheter, jugulars/femoralis/subclavia catheter). The maintenance bundle consists of: proper daily nursing care, preparation of intravenous medication, administration of intravenous medication, blood drawing from a CVC, administration of a bacterial-static solution to short bowel patients, removal procedure, port catheter (e.g. general port catheter care, administration of medication and removal of the port needle).

Finally, once accepted the protocol will be made easily accessible at our in-hospital Intranet through keywords enabling to navigate to all sub themes. In addition we will write a CVC infection prevention manual with special attention to infection prevention in sick children and the side effects of BSIs. It will also present evidence from the literature on preventive interventions.

#### ***Barriers***

We have already explored barriers to successful implementation of the selected interventions. In five in-depth semi structured interviews with physicians and nurses we discussed the key elements of the CVC procedures that would have to be changed – and at the same time elicited potential barriers. Nurses as well as physicians said that applying an air dry time of 30 seconds after disinfection of the skin or needless connector was time-consuming. Physicians in addition doubted the necessity to use a new needle after failed attempts to insert a CVC through the skin.

#### ***Measuring***

The outcome measure is the number of CA-BSIs per 1000 line-days, calculated by dividing the number of bloodstream infections by the risk-adjusted denominator 1000 line-days. As a baseline measurement we prospectively collected data from 1st January 2011 to 1st January 2012. Over that period the number of CA-BSIs per 1000 line-days was 11.2 at the neonatal intensive care unit and 7.9 at the pediatric intensive care unit.

As secondary outcome (process measures) we selected degree of adherence to safety procedures, e.g. maximum sterile barrier during the CVC insertion procedure and adherence to hygienic protocols during intravenous administration. Adherence will be documented with the use of two observation tools (Tables 3 and 4). Performance related to the insertion procedure will be measured with two tools: use of the time out procedure that is linked with the CVC insertion protocol (Additional file 1: Table S1) and a questionnaire daily popping up in the patient’s electronic record informing after signs of infection at insertion site as well as the need of having the CVC in place.

**Table 3:** Process indicators CVC insertion monitoring form

	**Process indicator**	**Satisfied**
1	Skin cleaned with alcohol 70% with chlorhexidine 0.2%; or chlorhexidine 0.2% in water in infants with a gestational age < 26 weeks	Yes O No O
2	Air dry of at least 30 seconds after skin preparation prior to guide-wire insertion	Yes O No O
3	New needle used after each attempt to insert a CVC through the intact skin	Yes O No O
4	Sterile drape covering the patient for least 80%	Yes O No O
5	The provider inserting the CVC used maximum sterile barrier e.g. sterile gown, double sterile gloves, mask, and hat	Yes O No O
6	Optimal insertion site was selected or another site was argued for	Yes O No O
7	Echo use during insertion of jugularis and subclavia catheter	Yes O No O
8	Chlorhexidine impregnated sponge used in infants < 40 weeks gestational age	Yes O No O

*CVC* central venous catheter.

**Table 4:** Process indicators CVC nursing care monitoring form

	**Process indicator**	**Satisfied**
1	Appropriate hand hygiene prior to preparation of intravenous medication	Yes O No O
2	Disinfection of the cap or ampoule’s surface using alcohol 70%	Yes O No O
3	Applying 30 seconds air drying time after disinfection ampoule’s surface	Yes O No O
4	Disinfection of connector with alcohol 70%^#^	Yes O No O
5	Or disinfection of the stop cock in case intravenous medication is administered using an extension-line applying alcohol 70%^#^	Yes O No O
6	Applying 30 seconds air dry time for the stop cock of connector	Yes O No O
7	Daily question whether the CVC is still needed in PDMS	Yes O No O
8	Daily check of the insertion place for inflammation	Yes O No O
9	Aspirated blood from the CVC will not be returned to the patient	Yes O No O
10	The blue side of the Biopatch is visible	Yes O No O

*CVC* central venous catheter, *PDMS* patient data management system.

^#^select one depending on the mode of administration.

#### ***Implementation strategies***

*Engage*. We will inform all stakeholders and senior management staff about the current high number of CA-BSIs per 1000 line-days and the need to reduce this number, and address the undesirability of having different CVC protocols in place – implying we need to implement general preventive measures hospital-wide. Representative stakeholders from all wards will be invited to join the steering committee. Final approval of the CVC protocol by the steering committee is required and the members will support implementation in their respective wards and operation room suite.

Table 5 provides an overview of proposed implementation strategies. Strategies for the dissemination of knowledge are based on the Pronovost-model and others [12,24,25].

**Table 5:** Selected implementation strategies

**Intervention types**	**Components**
Education program, theme week	Multidisciplinary seminar
	Workshop for physicians
	Instruction for nurses
	Instruction on hygienic insertion and maintenance
Reminders	Leaflets
	Screen savers
	Daily questionnaire CVC need, pop-up in electronic patient management system
	Sticker attached at disinfection solution: apply 30 seconds air dry time
Feedback (quarterly)	Reporting incidence of CA-BSI
	Reporting adherence to insertion bundle
	Reporting adherence to maintenance bundle
Engagement of the managing staff	One-liners combined with picture
Awareness	Time out procedure, and staff empowerment to stop in case of protocol violation
	Daily goal sheet
Procedures	Revised protocols
	CVC infection preventions manual.
	Timers
	30 seconds air dry timer displayed on bedside screen

*CA-BSI* catheter-associated bloodstream infection, *CVC* central venous catheter.

*Engage & educate*. To improve engagement and education level we will organise a hospital-wide theme week dedicated to CA-BSIs reduction. Events are aimed at educating all staff involved on the new CVC protocol and CVC infection prevention manual and persuading them to change their usual care and comply with the new protocol. Two weeks prior, posters announcing the program will be spread, provided with supportive one-liners from senior management and senior clinical leaders including their portraits [26]. The key players fully supporting the new protocol will act as role models for more junior colleagues. During the CVC week, flyers presenting the key players’ one-liners and the on-liner messages will be used as screen savers throughout the children’s hospital [27].

A tentative schedule is the following:

Monday; flyer distribution, informative mini-symposium, marking of ten potential dirty surfaces at each ward, attaching a sticker on them and culturing the surfaces.

Tuesday; nurses visit bacterial laboratory and the new guidelines are discussed;

Wednesday; physicians and nurse practitioners receive clinical instructions; tables in the staff restaurant are decked out with placemats asking questions on appropriate CVC care; lecture on reasons for non-adherence to hand hygiene protocols.

Thursday; clinical instructions and lecture to promote high adherence to documenting CVC data in the patient data management system.

Friday, theme week wrap-up with musical act during lunch-time in the staff restaurant and a closing ceremony.

Detailed information concerning the infection prevention week is supplemented at Additional file 2.

For educational purposes we will use several types of reminders, such as screen savers; stickers attached on disinfection solution bottles displaying the message: “apply 30 seconds air dry time”; infection prevention messages printed on toilet paper. Furthermore, if a CVC is in situ, a questionnaire will daily pop up in the patient’s record in the electronic patient data management system. The questionnaire addresses the following items: “Is the transparent dressing in good order?”; “Is there any redness near the insertion place?”; and “Is the CVC still needed?”. The response categories will be collected at nominal level e.g. yes or no.

*Evaluate*. Quarterly, the primary and secondary outcomes will be measured to evaluate whether the intervention is successful and to identify possible flaws. The information obtained may serve as input for additional improvements. Data will be compared to the baseline measurement data and data collected in the previous period, if applicable, by interrupted time series analysis. Findings will be reported to the directors of the children’s hospital. Furthermore, findings on ward level will be presented to the management of the relevant ward. For steering purposes we will make use of insightful graphs and tables. Successful outcomes may be an incentive to continue on the road we have taken; negative outcomes may be an incentive to increase efforts.

*Endure and extend*. Pronovost and colleagues later added this idem to their model [12]. We postulate that the daily questioning on the need to have the CVC in place, as sketched above, will create awareness of safety precautions. Furthermore, we presume that sharing of the quarterly performance outcomes will improve perceptions of safe patient care. Finally, encouraging open discussion about possible procedure flaws and potential improvements will benefit the CVC process at large [25].

### Primary outcome

The primary outcome will be the laboratory confirmed number of CA-BSIs per 1000 line-days, following the CDC definition: (1) one or more positive blood culture(s) (not skin flora) and no other infection source; or (2) one positive blood culture and clinical signs and no other infection source [28]. The diagnosis will be made jointly by a member of the research team (OH, CvdS) and an infection control practitioner. Discrepancies between these persons will be resolved through discussion until consensus is reached; or will be solved by an independent physician using the institutional microbiological database and the patient file. The CDC defines CA-BSI as a primary BSI if a CVC was in place 48 hours before the development of the BSI and if it is not bloodstream related to an infection at another site [29]. A CVC in situ is defined as a catheter introduced into a vein that terminates close to the heart or into one of the great vessels, and which is used for infusion, blood sampling or hemodynamic monitoring. Data concerning the number of line-days will be abstracted from the digital patient data management systems. It may be an umbilical venous catheter, a percutaneous CVC, a tunneled catheter, a port, or a peripherally inserted central catheter (PICC) [30].

### Secondary outcomes

Rate of adherence to the CVC insertion bundle and rate of adherence to the maintenance bundle will serve as secondary outcomes.

The attending nurse will assess adherence to the CVC insertion bundle during the insertion procedure (Table 3). To this aim we designed a digital tab with drop-down menu for the two different patient data management systems. The time out procedure prior to the CVC insertion is predominantly added for creating safety awareness during insertion (Additional file 1: Table S1). During the insertion procedure all involved healthcare workers are encouraged to make comments and to stop the procedure in case of violation of insertion bundle. Intravenous medication preparation and administration items will serve to measure adherence to the CVC maintenance bundle (Table 4). Purpose-trained nurses will randomly assess these items during planned daily care.

### Economic costs and benefits

Additionally, the cost-effectiveness of the implementation of the CVC bundle will serve as a secondary outcome measure, to be established through cost-effectiveness analysis. Taking a health care perspective, the cost-effectiveness analysis will make a comparison between the pre-intervention period ('usual care’) and the post-intervention period regarding both the total costs and the number of CA-BSIs per 1,000 line-days.

Among the costs included will be the direct medical costs relating to CA-BSIs. These costs will be calculated by multiplying resource utilization with a unit cost price. As much as possible, real economic cost prices will be used rather than charges. Cost prices will be calculated according to established methods [31]. The time horizon will be from the patient’s hospital admission until discharge.

Any possible savings on the costs of medical care need to be balanced against the costs of implementing the CVC bundle. All costs related to the implementation process will be taken into account. This implies that the study will take account of the costs for the implementation strategies (Table 5), as well as the changes in the care implicit to the implementation of the CVC bundle (Table 2) insofar as they result in additional costs compared to usual care. To arrive at total costs, we will include both personnel costs, material costs, and overhead costs.

### Data collection

Four nurses trained in observation will unobtrusively observe staff when they apply the insertion and maintenance bundles. Observation will be guided by a self-designed structured case report form. The staff will be unaware of the reason for the observations; they are already frequently observed for training or research purposes. Data collection will be at random moments seven days a week and 24 hours a day. Sealed envelopes will be used to ensure an equal allocation of these two-hour observations over the wards and observation times during the day. Data on sex, (birth) weight, and age of the patients involved will be retrieved from the patient data management systems (Additional file 3: Table S2). Disease severity of infants admitted to the neonatal intensive care unit will be documented by the Clinical risk index for babies (CRIB) score [32]; that of children admitted to the pediatric intensive care unit by the Pediatric Risk of Mortality III score (PRISM III) [33] or Pediatric Index of Mortality (PIM) score [34]; that of children admitted to the oncology department by the Lansky score (children ≤ 9 year) [35] or Karnofsky score (children > 10 year) [35]. There is no suitable disease severity score for patients admitted to the medium care ward, due to the diversity of diseases.

### Statistical analysis

Data will be expressed as median and interquartile range (IQR), unless indicated otherwise. The baseline data showed that 69 children developed a CA-BSI. The effect of the intervention on rate reduction will be determined with a segmented loglinear regression analysis of interrupted time series, using a pre-intervention, intervention, and post-intervention segment. The slope or trend of the segments indicates the rate of change in time. An abrupt change at the time of the implementation indicates an immediate effect. Introducing slopes (percentual changes in time in infection rate before and after the implementation) corrects for unassociated background trends. A change in slope after the introduction of the intervention may identify a gradual effect of the implementation [17,36]. We aggregated CA-BSIs per 1,000 line-days over 3-month periods. This figure will also be used in the cost-effectiveness analysis, which is concerned with the differences in outcome and total costs between the pre-intervention and the post-intervention period. Cost-effectiveness is assessed through the calculation of an incremental cost-effectiveness ratio (ICER). The ICER is calculated as the incremental costs per case of CA-BSI per 1,000 line-days avoided. The cost-effectiveness analysis will include a sensitivity analysis to assess how sensitive the results are to any assumptions made.

### Sample size

For the power analysis we made some simplifying assumptions: only pre- and post-intervention periods are compared and instead of comparing slopes we compare levels of infection rate. Under these circumstances a length of 1.9 years before and after intervention (leading to an assumed number of 141 cases before) is enough to detect a drop in rate to 70%. When we apply loglinear regression analysis with Poisson error distribution corrected for number of days at risk we find that that two times 1.9 years leads to a power of 80%.

The baseline rates of adherence to the insertion and maintenance bundles are estimated at 60% and 20%, respectively; the post intervention rates aimed at are 100% and 45%, respectively. Sample size calculation indicated that each sequential period requires 15 insertion observations and 45 maintenance observations to detect a relative difference of 37% and 125% alpha of 0.05 and a 1-beta of 0.8.

### Ethical aspects

The Erasmus MC Institutional Review Board approved this study (MEC-2012-375).

## Discussion

The implementation strategy encompasses a multifaceted program tailor made for this specific hospital and ready for use. We hypothesize that implementation of this program will result in fewer CA-BSIs and improved adherence to CVC bundles [5,22,24,37]. Tailored implementation strategies – i.e. based on content analysis of barriers and facilitators – seem to be more effective than non-tailored strategies [38,39]. The Pronovost-model effectively helped to design strategies from the first to the last phase.

Various strategies will be employed. First, education by means of a mini-symposium, workshops, presentations, and so on. Second, improving staff intrinsic motivation, e.g. by audit, feedback, and reminders. Third, organisational change: preparing on single protocol for all patients. Fourth, use of ICT: e.g. a timer on the computer screen at the bedside to clock the 30 seconds air drying time, online access to guidelines and the CVC infection prevention manual, use of screen savers, and daily reminder informing whether the CVC is still needed. Fifth, feedback: quarterly reporting of the CA-BSI incidence and the rates of adherence to the insertion and maintenance bundles. Sixth, awareness: time out procedure and daily goal sheet. Seventh, engagement of the managing staff: showing their commitment to the aim.

The Pronovost-model recommends enlisting all local stakeholders involved in patient care and discussing with them potential barriers and facilitators to adherence to the developed protocol. The Pronovost-model does not provide for eliciting support from senior management of the hospital. Nevertheless, they will be asked to demonstrate their commitment and assume ownership of the general aim to reduce CA-BSIs hospital-wide [25]. We will explicitly designate senior management as ambassadors of the goals [40] by publishing their one-liners and portrait pictures. These ambassadors should convince all healthcare workers that safer CVC care is an important goal and make clear they support the campaign. The senior management is officially accountable for patient safety and even may act as role models.

Use of opinion leaders will be added to the implementation strategy. Clinical and senior management need to show their vision and clearly dissimilate this particular aspect of safety culture. Senior management of effective infection prevention programs dissimilated their success as improved clinical excellence and inspired their staff [26]. Furthermore, senior management will help resolve organizational and financial barriers and practically support initiatives [25,26].

The Pronovost-model describes the implementation process in broad terms; development of a fitting protocol or work-instruction is not included [12]. We will therefore add a comprehensive description of how to develop a work-instruction. Evidence from recently published studies should support the newly developed protocol and providing evidence will perfectly fit into phase 1 of the Pronovost-model; building evidence in favour for the chosen intervention.

Feedback is a widely used, powerful measure to increase adherence to infection prevention measures such as good hand hygiene [24,41]. However, once feedback is stopped any unwanted behaviour could come up again [24,42]. On the other hand, providing continuous feedback is time consuming and therefore not realistic. Feedback should be used firstly to alter initial unwanted behaviour and this should ideally move into desired behaviour as an intrinsic driven and well-conditioned behaviour.

Education is often used to support behavioural change, especially if flaws in knowledge are observed [5,40,43]. A complicating factor is that level of knowledge varies among healthcare workers categories and within categories. This should be borne in mind when developing a hospital-wide education program. Physicians may tend to appreciate knowledge more than do nurses, and evidence-based education could be very useful to promote physicians’ desired behaviour [44]. On the other hand, a wash-out effect is often observed [45,46]. This means that knowledge previously received may recede to the background. Ideas on what is effective in infection prevention are developing over time, so regular updates are essential. In addition, repetition of education programs is necessary in a teaching hospital like ours; many healthcare workers are in training and leave the hospital after having completed their education program.

The Pronovost-model is merely medical oriented. The main point of departure from the model is knowledge transfer, which fits into physicians’ learning style [44]. Goossens et al. found that strong scientific evidence was the strongest determinant of physicians’ behaviour [44]. However, regarding our goal, a clear healthcare team angle seems to be more appropriate for a broad dissimilation of improved hygienic behaviour among all members of the multidisciplinary team. Nurses have a more active learning style, and the strongest determinant of knowledge acquisition was found the be the fact whether the subject was 'interesting […] or not’ [44]. This phenomenon affects whether a new procedure is potentially beneficial for patients or gets embedded in the daily care.

We postulate that the proposed study has methodological strength because it is guided by a validated implementation model that has been translated into a hands-on program and is described in detail for implementation hospital-wide. Furthermore, regularly reporting the outcomes is in line with the ORION statement promoting transparent reporting on intervention studies aimed to reduce nosocomial infections. ORION recommended interrupted time series as preferable method for analysis showing the change in results over time.

We anticipate several challenges in this study. (1) Effectiveness of interventions is preferably evaluated by randomisation into an intervention and control group. However, this method is inappropriate for our aim to implement an intervention hospital-wide, and the interrupted time series design is the second best solution and in line with the ORION guidelines (2). The CVC bundle is adapted and tailor made for our hospital. Although this results in less generalizability, this naturalistic approach could help develop practical implementation strategies for other hospitals or other interventions. (3) The effective ingredients of the bundle are still unclear. However, as some interventions have been shown to be effective it would be unethical to test all separate interventions. (4) To control for confounders is a challenge due to the different safety climates in the different departments. By establishing a clear leadership we will try to show the benefits of a univocal approach towards infants’ CVC care.

Nevertheless, this detailed implementation strategy of the CVC bundle has a potential to effectively modify healthcare workers behaviour and reduce the number of CA-BSIs hospital-wide.

## Abbreviations

CA-BSI: Catheter-associated bloodstream infections; CDC: Centers for disease control and prevention; CVC: Central venous catheter; IQR: Inter quartile range; IV: Intra venous; ITS: Interrupted time series.

## Competing interests

The authors declare that they have no competing interests.

## Authors’ contributions

This manuscript was prepared by the authors. OH, EI and CvdS were responsible for the research question, OH and EI designed the study. OH and EI wrote the initial draft of the manuscript and all authors contributed to redrafting. CL is the statistician who offered advice on the power calculation, the sample size considerations and advice on the statistical methods. MP is a health scientist and provided economic evaluation advice. DT and RW are the general supervisors of the study and were involved in revising the manuscript. All authors read and approved the final version of the manuscript.

## Pre-publication history

The pre-publication history for this paper can be accessed here:

http://www.biomedcentral.com/1472-6963/13/417/prepub

## Supplementary Material

Additional file 1: Table S1 Time out procedure performed prior to CVC insertion. Click here for file

Additional file 2 Detailed program of the CVC bloodstream infection reduction theme week. Click here for file

Additional file 3: Table S2 Infants’ clinical characteristics Case record form. Click here for file
