# Supplementary material for: Implementation of a children’s hospital-wide central venous catheter insertion and maintenance bundle
Source: BMC Health Serv Res. 2013 Oct 14;13:417. doi: 10.1186/1472-6963-13-417 (PMC3853717; doi:10.1186/1472-6963-13-417)
Supplement: Additional file 1: Table S1 — Time out procedure performed prior to CVC insertion. [file 1472-6963-13-417-S1.doc]

Additional file 1: Table S1. Time out procedure performed prior to CVC insertion

Time out procedure Patient ID/ sticker

Insertion of CVC

ICK / ICN/ MC Unit:

Date: …………….

| Stop the procedure as soon as a ‘NO’ is noted,  Take care to correct the flaw and change this into ‘YES’,  Document in third column | YES | NO | Yes, was No |
| --- | --- | --- | --- |
| ID control of patient: |  |  |  |
| Name, sex and birth date | O | O | O |
|  |  |  |  |
| Attendees: |  |  |  |
| Attendees present themselves and name their functions | O | O | O |
| Hand disinfection with hand alcohol | O | O | O |
| Physician or nurse practitioner applied maximal sterile barrier:  (Sterile gown, sterile gloves, mask, and hat) | O | O | O |
| Insertion-cart including CVC is complete and covered with sterile drape according to the protocol | O | O | O |
| Correct size of CVC is present | O | O | O |
|  |  |  |  |
| Patient: |  |  |  |
| Patient is sufficiently sedated on the guidance of the COMFORT behaviour scale | O | O | O |
| Additional sedatives are available |  |  |  |
| Analgesics are available | O | O | O |
| Age-appropriately monitored | O | O | O |
| The insertion area (insertion cart, patient, and place for MD or nurse practitioner) is lined with barrier tape | O | O | O |
| Patient is covered with sterile drape for at least 80% | O | O | O |
| Optimal insertion site selected by protocol | O | O | O |
| Skin disinfection by protocol | O | O | O |
| At least 30 seconds air dry time before introducing guidewire | O | O | O |
| A new needle at each attempt | O | O | O |
| CPR-form present (compulsory at ICUs) | O | O | O |

Date: …-………-………

Name and initials of pediatrician / nurse practitioner:

Name and initials of attending nurse:
