# Supplementary material for: Implementation of a children’s hospital-wide central venous catheter insertion and maintenance bundle
Source: BMC Health Serv Res. 2013 Oct 14;13:417. doi: 10.1186/1472-6963-13-417 (PMC3853717; doi:10.1186/1472-6963-13-417)
Supplement: Additional file 2 — Detailed program of the CVC bloodstream infection reduction theme week. [file 1472-6963-13-417-S2.docx]

**Additional material.**

Additional file 2: Detailed program of the CVC bloodstream infection reduction theme week.

*Monday.* Distribution of flyers including the theme week program at a glance, one-liners of senior management staff and leading clinicians, and the 10 key items that will be changed or need extra attention. The senior management staff will visit all wards to inform the healthcare workers about the need for prevention and remind them of the theme week. Coffee mugs printed with infection prevention messages will be spread, as well as petit fours depicting the theme week logo.

Healthcare workers will be asked to rank the most contaminated surface of their wards by attaching a red sticker. The infection control practitioner will culture these surfaces. Culture results will be presented at the theme week’s closing day. The person who selected the most compromised surface will receive an award.

The afternoon features a mini-symposium for all healthcare workers involved. It will introduce the rationale of the CVC theme week, e.g. observed suboptimal hygienic performance and need for changing hygienic behaviour, supported by evidence from recently published studies. The advantages of the state-of-the-art CVC protocol will be presented, e.g. tailored to the needs of patients, as well as the process and outcome measurements, expected changes for physicians and nurses, and recording of the presence of the CVC in the patient data management systems.

*Tuesday.* The following educational activities are planned on Tuesday, Wednesday, and Thursday: daily tours at the bacteriological laboratory explaining the processing blood cultures and identification of pathogens.

During lunch time all tables in the staff restaurant are decked out with placemats on which are printed 12 questions on CVC infection prevention and the following open question: do you have a solution for the problem that healthcare workers fail to take 30 seconds air dry time. A winner drawn from the correct entries will receive an award during the closing ceremony. In the afternoon nurses will be lectured on aspects of the CVC insertion and maintenance bundles that need extra attention.

*Wednesday.* Instruction lessons for physicians and nurse practitioners at different wards on improved hygienic procedures during CVC insertion. In the afternoon they are lectured on measures to improve adherence to hand hygiene protocols.

*Thursday.* An afternoon lecture on technical aspects of the documentation and the need for accurate CVC registration.

*Friday.* Musical act during lunch-time in the staff restaurant. A cabaret group will compose a cheerful song for this occasion, entitled: 30 seconds are needed. During the closing ceremony this week’s events are summarized, the results of the cultures are announced, and the questions on the placemats are discussed.
