# Supplementary material for: Implementation of a children’s hospital-wide central venous catheter insertion and maintenance bundle
Source: BMC Health Serv Res. 2013 Oct 14;13:417. doi: 10.1186/1472-6963-13-417 (PMC3853717; doi:10.1186/1472-6963-13-417)
Supplement: Additional file 3: Table S2 — Infants’ clinical characteristics Case record form. [file 1472-6963-13-417-S3.doc]

Additional file 3: Table S2. Infants’ clinical characteristics Case record form

| General information |  |
| --- | --- |
| Name observer | ____________________ |
| Date | __ /___ /____ |
| Time | __ : __ |
| Department/ unit | ________ /___________ |
| Elective or rescue patient care | E R |
|  |  |

| Patient characteristics |  |
| --- | --- |
| Patient ID | (place patient sticker here) |
| Male/ female | ________________ |
| Gestational age ^a^ (weeks) | ________________ |
| Age ^b^ (years) | ________________ |
| Birth weight ^a^ (gram) | ________________ |
| Weight ^b^ (kilogram) | ________________ |
| Disease severity (select one) |  |
| Clinical risk index for babies ^a^ (CRIB) | ________________ |
| Pediatric Risk of Mortality III score ^b^ (PRISM III) | ________________ |
| Pediatric Index of Mortality ^b^ (PIM) | ________________ |
| Lansky score ^c^ (children < 9 year) | ________________ |
| Karnofsky score ^c^ (children > 10 year) | ________________ |
|  |  |

^a^ for infants

^b^ for children

^c^ for children diagnosed with cancer
